# A Retrospective Cross Sectional Study of the Effectiveness of a Project in Improving Infant Health in Bwindi, South Western Uganda

**DOI:** 10.3389/fpubh.2018.00290

**Published:** 2018-10-12

**Authors:** S. Robert Kamugisha, Andrew E. Dobson, Alex G. Stewart, Nahabwe Haven, Birungi Mutahunga, Ewan Wilkinson

**Affiliations:** ^1^Church of Uganda Bwindi Community Hospital, Kinkizi Diocese, Kanungu, Uganda; ^2^College of Life and Environmental Science, University of Exeter, Exeter, United Kingdom; ^3^Institute of Medicine, University of Chester, Chester, United Kingdom

**Keywords:** SORT IT, operational research, community nurse, community health volunteer, community health worker, infant health

## Abstract

**Introduction:** Low-cost community-based interventions to improve infant health potentially offer an exciting means of progressing toward the Sustainable Development Goals (SDGs). However, the feasibility of such interventions in low-income settings remains unclear. Bwindi Community Hospital (BCH), Uganda implemented a 3-year nurse-led community project to address child-health issues. Nurses supported Community Health Volunteers (CHVs) and visited mothers pre- and/or postnatally to assess and educate mothers and infants. CHVs gathered data and gave basic advice on health and hygiene to mothers. We hypothesized that increased interventions by nurses and CHVs and increased contact with households, would improve health and reduce infant mortality.

**Methods:** This was a retrospective cohort study analyzing routine data of all children born between January 2015 and December 2016. There were three interventions: antenatal nurse visit, postnatal nurse visit and CHV participation. Children received different numbers of interventions. We defined four diverse outcomes: facility-based delivery, immunization completeness, nutritional status, and infant mortality. Odds ratios, adjusted odds ratios, and multivariate logistic regression were used to assess associations between interventions and outcomes.

**Results:** Of the 4,442 children born in 2015 and 2016, 91% were visited by a nurse (81% antenatally and 10% postnatally); 7% lived in villages with a high participating CHV. Households receiving a postnatal visit were more likely to complete immunization (aOR: 1.55, *p* = 0.016) and have the infant survive (aOR: 1.90, *p* = 0.05). Children from a hard-to-reach village (no road access) were less likely to be delivered in a health facility (aOR: 0.55, *p* < 0.001) and less likely to survive in their first year (aOR: 0.69, *p* = 0.03). Having two or more interventions was associated with a child having all four positive outcomes (aOR 0.78, *p* = 0.03). Lack of baseline data, a control area, or integrated assessment data limited more detailed evaluation.

**Conclusion:** Visits to mothers after birth, by a nurse to educate and identify child illness, were associated with lower infant mortality and improved infant health as measured by completion of immunizations. Community health interventions could potentially have a greater impact if focused on hard-to-reach areas. Building evaluation into all project designs, whether local or internationally funded, would enable greater learning, and hence better use of resources.

## Introduction

### Preventing child deaths

Despite a global reduction of over 50 percent in preventable child deaths between 1990 and 2013, over 6 million children aged under 5 years died in 2013, across the world (1). Almost half (47.4%) these deaths occurred in 47 African countries. Only eight of these countries were on track to achieve the Millennium Development Goal target 4A—to reduce under-five mortality by two thirds between 1990 and 2015 (1).

In sub-Saharan Africa, in 2013, the neonatal mortality rate (NMR: probability of dying within 28 days of birth) was estimated at 31 deaths per 1,000 live births, 50% higher that the global NMR of 20 while the infant mortality rate (IMR) (the probability of dying before the first birthday) was estimated at 61 deaths per 1,000 live births, almost twice the Global IMR of 34 (2). Successive Uganda Demographic Health Surveys showed that the IMR dropped from 54 deaths per 1,000 live births in 2011 to 43 in 2016 (3, 4).

Around Bwindi Community Hospital (BCH) in southwest Uganda, the IMR remained high at 78 per 1,000 live births in 2013, with under five mortality rates at 109 per 1,000 live births (2012 household survey, unpublished data). These were both higher than the national rates of 43 and 64 per 1,000 live births, respectively (3).

The target of Sustainable Development Goal (SDG) 3 is to reduce the neonatal mortality (NMR) to at least as low as 12 per 1,000 live births and under-5 mortality to at least as low as 25 per 1,000 live births by 2030 (5). This will also reduce the IMR (2).

The leading causes of under-five mortality are neonatal deaths, pneumonia, diarrhea, malaria, measles, and AIDS (2). Many of these deaths occur in children whose immune system is already weakened by malnutrition.

The neonatal period is the most vulnerable time for child survival, with about 40% of deaths of children under five (6). Deaths in this period may often be due to preterm birth and complications during labor and delivery (2). Young mothers and births shortly after another sibling also increased the risks (2). All pregnant mothers and their babies should have access to high quality care during pregnancy, throughout birth and the postnatal period (5).

One of the major constraints identified in the achievement of SDG 3, to improve maternal health and reduce child mortality, was the shortage of skilled trained health workers in most low and middle income countries (6). In Uganda, as in most other African countries, this skills shortage, and the cost of accessing adequate health care, represent major obstructions to universal health care.

### The potential of community nurses and volunteers

In Uganda, as elsewhere, nurses working in the community are expected to screen and refer patients to health facilities as well as mobilizing communities for vaccination and community health activities (7). In addition, community health volunteers (CHV) can complement nurses by performing a range of activities including preventive counseling, health education, behavioral change and health promotion (8).

Lay community health workers, also termed community health volunteers, are crucial in complementing the roles of trained health staff, in improving health outcomes in pregnancy and in infants, (9–11) In a systematic review, community health interventions were associated with a reduction in neonatal mortality; the intervention group had an NMR of 43 compared to 49 deaths per 1,000 live birth in the control (9). Community Health Volunteers (CHVs )visiting pregnant mothers increased facility-based deliveries threefold and also boosted antenatal clinic visits in one Ugandan study; and mothers were much more timely in seeking care for neonatal illness (12, 13). Providing a vaccine-related educational message to mothers at home by trained CHVs has been shown to be an effective and practical strategy to improve childhood immunization rates in a low income setting (14). In another Uganda study, visits by CHVs were associated with a 5% reduction in the prevalence of underweight babies and a halving of the number of deaths in children under-5 (11).

### The intervention: bwindi community nurse-led health for all (HEAL) project

Recognizing that nurses and CHVs could improve maternal and child health outcomes, BCH implemented a 3-year nurse-led *Health for All* (HEAL) project in 2014 with funding from international donors. Community nurses worked in conjunction with community-based CHVs in households across the 101 villages in the BCH catchment area (population 71,000).

**Figure 1:**
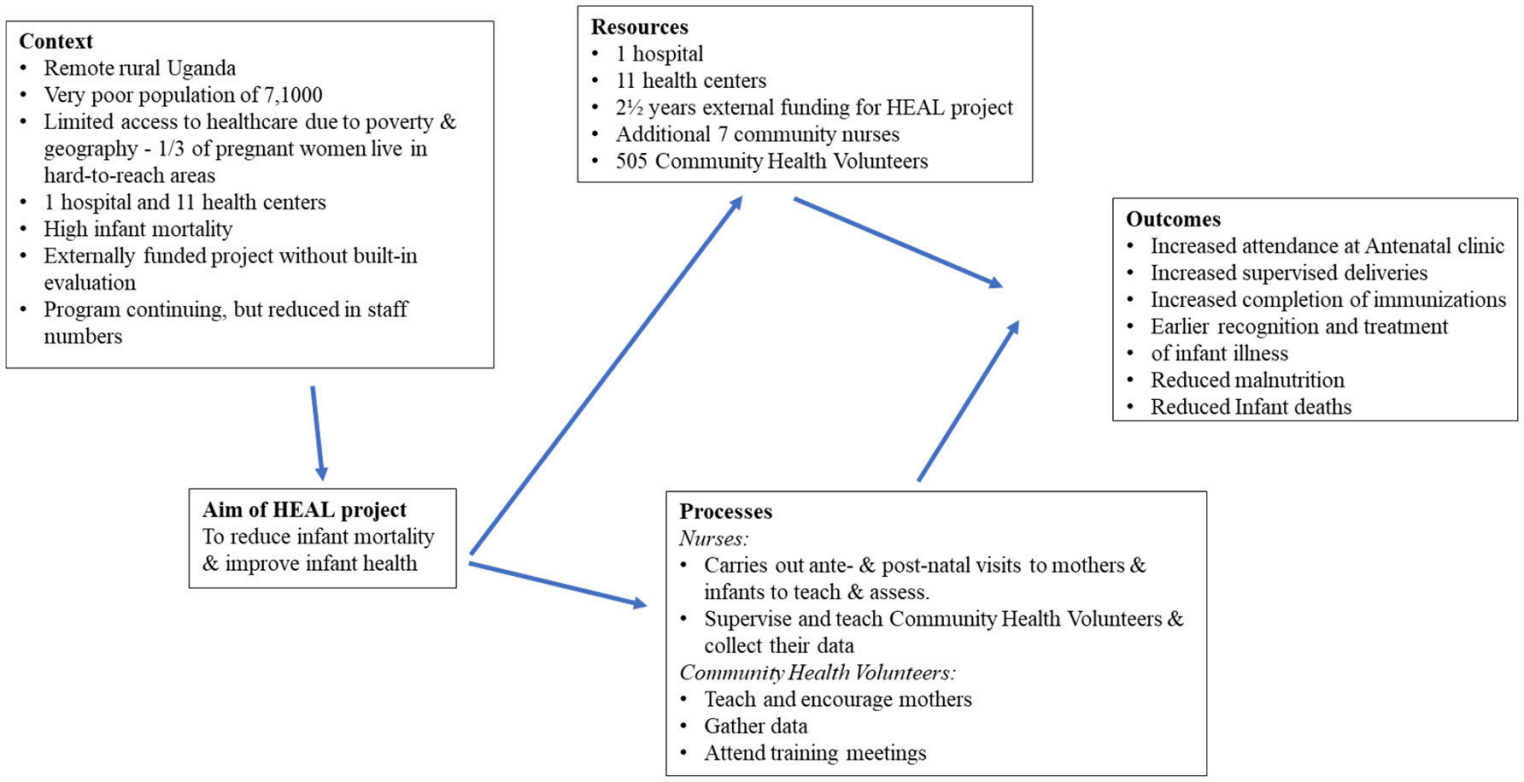
Conceptual framework for Health for All (HEAL) project at Bwindi Community Hospital, Uganda.

The conceptual framework is shown in Figure 1. In the context of remote rural Uganda, it was anticipated that the additional seven community nurses working with the HEAL project would reduce infant mortality by increased attendance at ante-natal clinics (ANC) and increased supervised deliveries, increased completion of immunizations by 1 year of age, earlier recognition, and treatment of infant illness and reduction of malnutrition and IMR.

An evaluation of this project is important because from 2017 onwards there will be only sufficient resources to support three rather than seven nurses in the field. Decisions have had to be made how to use the reduced resources to best effect.

The aim of this study was to determine whether the inputs from the HEAL project, the nurse visits antenatally and/or postnatally, combined with CHV visits to households, had an effect on the health (as defined by nutrition status, completion of immunizations by 9 months and where the child was delivered) or mortality of infants born in those households within the three sub counties of Kayonza, Kanyantorogo and Mpungu in Kanungu district, Uganda, between January 2015 and December 2016. Specific objectives were to: (i) examine the number and proportions of nurse-led and CHV interventions in all households, and in hard-to-reach areas, (ii) quantify four health outcomes of the children born in terms of:- facility based deliveries, completion of relevant immunizations, achievement of good nutritional status using mid-upper arm circumference, and survival beyond 1 year of age, and (iii) explore potential associations between nurse-led and CHV interventions and children's health outcomes.

### Interventions details

The HEAL project started in 2014 in Kanungu district. Seven nurses and 500 CHVs in the 101 villages of the three sub-counties worked together, complementing each other's roles, in a planned and systematic way. Each day a community nurse met CHVs in the CHVs own villages to discuss a health topic, collect data, and visit pregnant women and newborn infants identified by CHVs.

The nurse-led and CHV interventions documented for the study included (a) a visit by a nurse to the pregnant woman before child birth, (b) a visit by a nurse to the family of any neonate considered to be at risk by the CHV, and (c) home visiting activity by the CHV to the household to see the mother and child before birth and after delivery.

The CHVs' role was to collect data on the number and location of pregnant women, identify women who had given birth and any deaths within the village households and visit households to check the immunization coverage of those under 1 year old, screen for malnutrition using mid-arm circumference, and refer children and others to health facilities where necessary. They also educated households about child and maternal health and health-promoting behaviors including nutrition [using pictorial counseling cards (15)], sleeping under an insecticide treated bednet, and encouraged mothers to make appropriate use of local health facilities, including attending antenatal clinics and health facility deliveries.

During antenatal visits, the nurses discussed available preventive services, care during pregnancy, birth preparedness, and danger signs during pregnancy. For postnatal visits, the nurses discussed immediate care for the new born, newborn danger signs, examined baby for abnormalities, and referred any sick newborn to health facilities. Selection of households to be visited by the nurses was decided in discussion with the CHV.

The HEAL project was a donor-funded project. During the project, some households received a full set of nurse and CHV interventions, and others only some or no interventions. This study, therefore, focused on assessing whether the full package of interventions or just one, two, or three interventions were associated with improved childhood outcomes.

## Methods

### Study design

This was a retrospective cross-sectional study using routinely collected data.

### Setting

BCH is a not-for-profit hospital run by the Church of Uganda, and is located in South Western Uganda, Kanungu District a 12-h drive from Kampala, the capital city. The area has a rugged topography with narrow valleys intersected by rivers and steep hills (16).

Its total catchment area includes more than 100,000 people, most being subsistence farmers living on < 1 dollar per day.

The poor road network means that a 1/3 of children live in remote, hard-to-reach villages.

### Study population

All children born in households of the 101 villages of Kayonza, Mpungu, and Kanyantorogo (population 71,000) between January 2015 and December 2016 were included in the study.

### Data variables, data sources, and data collection

The independent variables collected were the number of nurse-led pre-birth visits; number of nurse-led visits to assess the neonate; a household in a hard to reach area; CHV participation level [defined as “high” if the CHV (a) attended ≥75% of expected monthly meetings, and (b) visited his/her households and collected / submitted ≥75% of expected monthly reports on pregnant women, newborn babies, and sick members of his/her catchment households, and (c) screened and monitored ≥75% of the children under-5 years of age in his/her catchment households; (17)].

There were four dependent variables collected: child delivered in a health-facility; complete set of immunizations (see below); good nutritional status defined as mid-upper arm circumference >18.5 cm (18); and death of the child before 12-months of age.

Hard to reach households were defined as being in a village more than 5 km from a health facility, whose access was up the side of a ridge over 500 m high, and which could not be accessed using a motorcycle because of the lack of a passable track.

Facility-based delivery was used as a proxy to measure skilled care during delivery [as recommended in the SDG (5)]. Complete immunization, according to the Uganda National Expanded Program for Immunization, was defined as a child having received Bacillus Calmette–Guérin (BCG) vaccine, oral polio or inactivated polio vaccine (OPV/IPV), diphtheria, pertussis, tetanus, *Haemophilus Influenza* Type b (Hib), hepatitis B, conjugate pneumococcal and measles vaccines before the end of the first year of life (7).

The CHV and nurses recorded details of household visits and childhood outcomes in a book, and were entered an electronic database maintained at BCH. Any missing electronic data was subsequently retrieved from the nurses' books, by the principal investigator between November 2017 and January 2018.

### Analysis

Data validation of the nurses' records was carried out at the point of data entry into the electronic system and during project monitoring during the lifetime of the project. Electronic data was exported into EpiData (version 4, EpiData Association, Odense, Denmark).

Odds ratios were calculated for each individual outcome variable, in relation to each independent variable, and tested for statistical significance using the chi-square test. Multivariate logistic regression was used in the standard way to calculate adjusted odds ratios and to recalculate *p*-values in order to test for variables that may have been confounding the bivariate associations observed between an individual independent variable and an outcome variable (19).

Inclusion of variables in this logistic regression was done on the basis of significance levels (i.e., all independent variables for which the bivariate chi-square test of association with the outcome variable produced a *p*-value of <0.2 were included for that outcome variable, as previously mentioned), as well as the pragmatic availability of data. In other words, the study was limited to the availability of three intervention variables and one possible confounder. The ratio of sample size to the number of independent variables (sample size of 4,442 and four independent variables) was comfortably higher than the 10–20 minimum generally recommended (20, 21).

As all the variables being used were binary variables, and at most three independent variables were used in any calculation, this was a simple use of logistic regression, e.g., free from the potential distorting effect of outliers. The independent variables were checked for collinearity. There was some correlation between the variables for postnatal nurse visits and CHV participation, and between CHV participation and living in a hard-to-reach village, which will have widened the reported confidence intervals and increased *p*-values. Where three independent variables were used, a sensitivity check was done on the results for whether the coefficients and *p*-values for the two variables with lowest *p*-values were sensitive to the inclusion or removal of the third variable from the model. In neither case were they sensitive.

Levels of significance were set at 5%.

### Ethics

Ethical clearance was obtained from the research and ethics committee Bwindi Community Hospital, Kanungu, Uganda and also from the Ethics Advisory Group of International Union Against Tuberculosis and Chronic Lung Disease, Paris France.

## Results

### Household characteristics and Nurse-Led/CHV interventions

**Figure 2:**
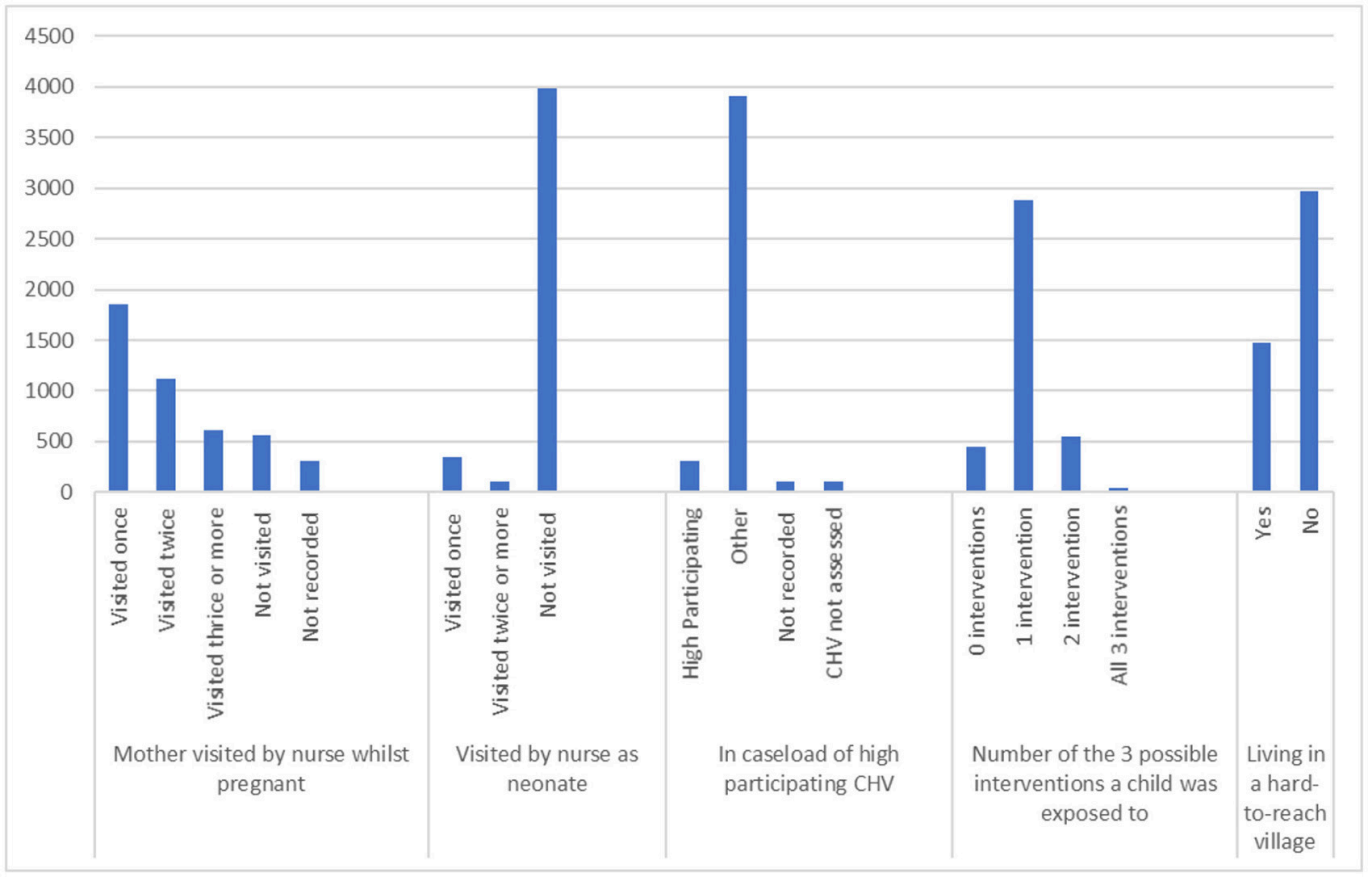
Numbers of households who had 0–3 of the 3 study interventions, and numbers of households in a “hard to reach area” in Bwindi area, Uganda.

There were 4,442 children born during the study period, one third (1,474) in households in hard-to-reach areas. The numbers and proportion of households visited by a nurse before or after delivery, visited by high participating CHV, those receiving 0 to 3 interventions, and those in hard-to-reach areas are shown in Figure 2. There were 3,577 (81%) households visited by a nurse during the pregnancy, while 454 (10%) neonates were also visited by a nurse. All household were visited by a CHV, and 314 (7%) infants lived in households visited by CHVs whose participation was rated as “high.”

### Childhood outcomes

Childhood outcomes for the study period are shown in Table 1. There were 3,650 (82%) births in a health facility, 3,800 (86%) infants had a complete set of immunizations before 1 year of age, 3,671 (83%) infants had normal nutrition and 4,269 (96%) infants were alive at 12 months. Out of all the children born during the study period, 2,586 (58%) had all four positive outcomes (delivered at a health facility, complete immunizations, normal nutrition, and survival to 12 months of age).

**Table 1:** Outcomes for all children born in 2015 and 2016 in Bwindi area, Uganda.

**Childhood outcomes**	***N***	**(%)**
All children	4,442	(100)
**DELIVERED IN A HEALTH FACILITY**		
Yes	3,650	(82)
No	484	(11)
Not recorded	308	(7)
**IMMUNIZATION STATUS**		
Complete	3,800	(86)
Incomplete	642	(14)
Not recorded	0	(0)
**NUTRITION STATUS**		
Normal (MUAC green) –≥18.5 cm	3,671	(83)
Mild malnutrition (yellow)	23	(< 1)
Severe malnutrition (red)	6	(< 1)
Not recorded	742	(17)
**INFANT STATUS AT 12 MONTHS**		
Alive	4,269	(96)
Dead	173	(4)
Not recorded	0	(0)
CHILDREN WITH A POSITIVE OUTCOME*		
Children with no positive outcomes	0	(0)
Children with 1 positive outcome	5	(< 1)
Children with 2 positive outcomes	95	(2)
Children with 3 positive outcomes	765	(17)
Children with 4 positive outcomes	2,586	(58)
Children without all 4 outcomes recorded	991	(23)

*MUAC, Mid-upper arm circumference*.

* *Facility based delivery, full immunization, well nourished, alive*.

### Associations between household characteristics, nurse-led and CHV interventions, and childhood outcomes

The results of associations between household characteristics, nurse-led/CHV interventions and childhood outcomes are shown in Table 2. With respect to delivery in a health facility, households from hard to reach areas had slightly fewer deliveries at a health facility compared with other households 83.9 vs. 90.5% OR 0.55 (CI 0.45–0.67; Table 2A). Having a nurse-led visit to the neonate was associated with more complete immunizations 90.3 vs. 85.0% OR 1.64 (CI. 1.19–2.27; Table 2B). There were no significant associations between any interventions and nutritional status (Table 2C). Better infant survival at 12-months was significantly associated with having a nurse-led visit to the neonate almost two-fold higher OR 1.89 (CI 0.99–3.61) and coming from an area that was not hard-to-reach (Table 2D) OR 0.70 (CI 0.52–0.96).

**Table 2:** To assess any associations between the nurse/community health volunteer interventions and the outcomes for infants, in 2015 and 2016 in Bwindi area, Uganda.

**Intervention/Geographical characteristic**	**Total no. of infants**	**No. with positive outcome [*N* (%)]**	**Unadjusted Odds Ratio (OR) (95% CI), *p-*value (chi-square)**	**Adjusted OR, *p*-value (logistic regression)**
**(A) OUTCOME 1: DELIVERY IN A HEALTH FACILITY**				
**Pre-birth visit**				
No	557	501 (89.9)	1	1
Yes	3,577	3,149 (88.0)	0.82 (CI: 0.61–1.10) *p* = 0.19	0.83 *p* = 0.24
**Neonatal visit**				
No	Not	Applicable		
Yes				
**CHV participation**				
Other	3,645	3,208 (88.0)	1	1
High participation	286	263 (92.0)	1.56 (CI: 1.01–2.41) *p* = 0.05	1.41 *p* = 0.13
**Hard to reach area**				
No	2,765	2,501 (90.5)	1	1
Yes	1,369	1,149 (83.9)	0.55 (CI:0.45–0.67) *p* < 0.001	0.55 *p* < 0.001
**(B) OUTCOME 2: IMMUNIZATION COMPLETENESS**				
**Pre-birth visit**				
No	557	485 (87.1)	1	
Yes	3,577	3,122 (87.3)	1.02 (CI:0.78–1.33) *p* = 0.89	
**Neonatal visit**				
No	3,988	3,390 (85.0)	1	1
Yes	454	410 (90.3)	1.64 (CI:1.19–2.27) *p* = 0.002	1.55 *p* = 0.016
**CHV participation**				
Other	3,911	3,363 (86.0)	1	1
High participation	314	259 (82.5)	0.77 (CI:0.57–1.04) *p* = 0.09	0.84 *p* = 0.33
**Hard to reach area**				
No	2,968	2,528 (85.2)	1	
Yes	1,474	1,272 (86.3)	1.10(CI:0.92–1.31) *P* = 0.32	
**(C) OUTCOME 3: GOOD NUTRITION STATUS**				
**Pre-birth visit**				
No	438	436 (99.5)	1	
Yes	3,013	2,992 (99.3)	0.65 (CI: 0.15–2.80) *p* = 0.56	
**Neonatal visit**				
No	3,308	3282 (99.2)	1	
Yes	392	389 (99.2)	1.03 (CI:0.31–3.41) *p* = 0.97	
**CHV participation**				
Other	3,274	3,247 (99.2)	1	
High participation	275	273 (99.3)	1.14 (CI:0.27–4.80) *p* = 0.86	
**Hard to reach area**				
No	2,473	2,455 (99.3)	1	
Yes	1,227	1,216 (99.1)	0.81 (CI:0.38–1.72) *p* = 0.58	
**(D) OUTCOME 4: INFANT SURVIVAL**				
**Pre-birth visit**				
No	557	538 (96.6)	1	
Yes	3,577	3,449 (96.4)	0.95 (CI:0.58–1.55) *p* = 0.84	
**Neonatal visit**				
No	3,988	3,825 (95.9)	1	1
Yes	454	444 (97.8)	1.89 (CI: 0.99–3.61) *p* = 0.05	1.90 *p* = 0.05
**CHV participation**				
Other	3,911	3,765 (96.3)	1	
High participation	314	301 (95.9)	0.90 (CI: 0.50–1.60) *p* = 0.72	
**Hard to reach area**				
No	2,968	2,866 (96.6)	1	1
Yes	1,474	1,403 (95.2)	0.70 (CI: 0.52–0.96) *p* = 0.03	0.69 *p* = 0.03

*CI, confidence interval*.

The results of associations between individual household characteristics and nurse-led / CHV interventions and having all four positive childhood outcomes together are shown in Table 3. When the interventions were analyzed individually, a nurse-led visit to the neonate was associated with a higher chance of all four positive outcomes OR 1.39 (CI 1.08–1.82; Table 3A). However, when the interventions were analyzed in combination, having two or three interventions together OR 0.77 (CI 0.61–0.96), or living in a not hard-to-reach area, were associated with having all four positive outcomes OR 1.26 (CI 1.07–1.48; Table 3B).

**Table 3:** Assessing if there are any associations between the interventions by nurses or Community Health Volunteers, where the infant lived and whether a child had all 4 health outcomes positive, in 2015 and 2016 in Bwindi area, Uganda.

**Intervention/Geographical characteristics**	**Total number of infants**	**Number with all 4 outcomes positive [*N* (%)]**	**Unadjusted Odds Ratio (95% CI), *p*-value (chi-square)**	**Adjusted OR, *p*-value (logistic regression)**
**(A) FOR THE 3 INTERVENTIONS TAKEN INDIVIDUALLY**				
**Pre-birth visit**				
No	438	337 (76.9)	1	
Yes	3,013	2,249 (74.6)	0.88 (CI: 0.39–1.12) *P* = 0.30	
**Neonatal visit**				
No	3,068	2,279 (74.3)	1	1
Yes	383	307 (80.2)	1.39 (CI: 1.08–1.82) *p* = 0.01	1.44 *p* = 0.01
**CHV participation**				
Other	3,051	2,275 (74.6)	1	1
High participation	253	199 (78.7)	1.25 (CI: 0.92–1.72) *p* = 0.15	1.17 *P* = 0.34
**Hard to reach area**				
No	2,301	1,758 (76.4)	1	1
Yes	1,150	828 (72.0)	1.26 (1.07–1.48) *P* = 0.005	1.26 *p* = 0.008
**(B) FOR THE 3 INTERVENTIONS TAKEN IN COMBINATION**				
**Number of interventions exposed to**				
0 or 1	2,784	2,064 (74.1)	1	1
2 or 3	520	410 (78.8)	0.77 (CI: 0.61–0.96) *p* = 0.02	0.78 *p* = 0.03
**Hard to reach area**				
No	2,301	1,758 (76.4)	1	1
Yes	1,150	828 (72.0)	1.26 (1.07–1.48) *p* = 0.005	1.31 *p* = 0.001

*CI, confidence interval*.

## Discussion

Our study suggests that selected community-based maternal, and neonatal care interventions delivered through nurses and CHVs were associated with reduction in IMR and an increase in immunization completion rates. Confirming that home visits by CHVs and nurses reduces infant deaths and increases immunization take up is important for many low and middle income countries. Wider implementation of this approach will help achieve SDG 3 in terms of reducing maternal and under-five mortality.

Living in a hard-to-reach area was associated with a reduced likelihood of delivery in a health facility and reduced probability of surviving the first 12 months.

### Comparisons with other findings

These results support other studies that have found that home visits by nurses working with CHVs in resource limited settings reduce infant deaths (11, 22, 23).

Elsewhere in Uganda, visits by trained CHVs who are able to contact nurses by mobile phone have been shown to increase health center deliveries and improve timely care-seeking for newborn illness (13), and to reduce deaths and illness in children under-5 (11). However, in our study all pregnant mothers and newborns were visited by the trained CHVs and it may be that the criteria used to assess if they were “high performing,” based on a previous study (17), were not the most relevant ones when it came to the effectiveness of their work with mothers and babies. In another Ugandan study (13), in the control area without the trained CHVs only 28% of deliveries were in health facilities, compared with 86% in the areas with the trained CHV. We did not find that visits by CHVs regarded as “high performing” made a significant difference when compared to other CHVs. As we had no control area in our study, the impact of the work of the CHVs in our study may be considerably underestimated, as even for those women resident in hard to reach areas, 84% delivered in health facilities.

Only 10% of mothers around Bwindi had a neonatal visit by a nurse. However, those infants were identified as being at risk by the CHV. An associated reduction in the IMR and an increase in completion of immunizations suggests it may be an effective approach.

A single antenatal visit, most commonly made by the community nurses, had no impact on child outcomes. This could be either because there really was no impact, or because the CHV visits also had a beneficial effect, or mothers made several visits to antenatal clinics, for which there was no data collected.

Being resident in a hard-to-reach area (not even accessible by motor-bike) was associated with a lower likelihood of delivery in a health facility, which is known to increase the risk of maternal and perinatal mortality (24). This is likely to reflect that these residents have more difficulty in accessing health care. A study in Zambia and Malawi found that the distance to health facilities in rural areas was a key factor explaining why most rural deliveries occurred at home (25). However in our population, 84% of mothers from a hard to reach area were delivered in a health facility, far more than the 32 and 52% quoted in the study from Zambia and Malawi (26).

“Hard to reach” is not just about distance, as in other topographies (24), but also includes the physical challenge of steep hillsides only accessible on foot, a major challenge.

Unsurprisingly, there was no measurable impact of the interventions on improving nutritional status. Nutritional status was generally good across the community, with < 2 percent of infants being malnourished, despite the underlying poverty. This may be due both to the nutrition education given by the CHVs and also to the relatively fertile soil and good local farming practices (27).

It is regrettable that evaluation was not built into the HEAL project from its conception. All projects, whether locally or internationally funded, should have a suitably designed data collection, including baseline data, built in to them to facilitate evaluation and hence maximize the learning from a project. Although generating additional cost, a formal evaluation would have greatly increased the learning from the project, thus ensuring appropriate care was provided, no harm was done, resources were not wasted, and informing decision-making when deciding if the project should be continue (28).

### Strengths

A major strength of the study was that it used data collected in an electronic dataset on all children born in the study community over the 24 months of the HEAL project. Furthermore, all births in the three districts were recorded, giving a large and complete dataset.

### Methodological limitations

This study had clear limitations. An evaluation was not planned into the project early and data collection was not ideal. A future study might prospectively include formal collection of baseline data plus data on attendance at antenatal clinics and timely seeking of care for illnesses in pregnancy or neonates. A control area would have made evaluation much easier and allowed assessment of the overall impact of the program, because the majority of the health centers that people attended are run by the Ugandan government rather than by BCH. This means that BCH does not automatically receive any electronic data from these health centers, only some manual data. An evaluation would have facilitated the sharing of knowledge of what works.

Our analysis assumed that hospital, health centers, community nurses and CHVs interventions each had a similar impact, as seen in the conceptual framework. In fact, a heterogenous effect is more probable.

The study only examined a package of care, not the constituent activities. For example, only counting the nurse visits does not give any indication of how they used their time or how they interacted with the CHVs and mothers. When there are several components to a program, it can be hard to attribute correctly which intervention led to which outcome. Nurses and CHVs are likely to be giving the same messages, and messages may need to be heard several times before mothers act on them. There is, therefore, likely to be a cumulative effect from the different aspect of the program. The program benefit may exceed the sum of the parts.

Our conceptual framework was perhaps limited by an emphasis on health care provision. A future study might include input from a wider range of disciplines, such finding ways to improve female education and increase incomes and financial security in a poor rural economy.

### Recommendations for research

1. Further exploration of the role of postnatal visits by a nurse in reducing the IMR and increasing completion of immunizations should be explored in BCH and elsewhere, ideally using a prospective design and control group.
2. Programs of community nurse and CHV visits to households should include routine collection of a comprehensive dataset to enable easy evaluation. Furthermore, good data collection in electronic databases should be seen as good practice in low and middle income countries as well as in high income countries.
3. In order to increase the proportion of mothers from hard to reach areas delivering under skilled supervision (as well as to reduce the risk of the newborn child dying), an assessment of the impact of a waiting hostel that reduces travel in labor for pregnant women should be made. There is one health facility in the area with such a waiting hostel.

## Conclusion

This retrospective analysis provides further evidence to suggest that the work of community nurses and CHVs has made a measurable difference to infant health in western Uganda. Future studies might usefully build evaluation into the initial program design to enable a more effective assessment of the effects on infant health. This would have made it easier to know how to continue the program with limited resources.

Wider implementation of home visits by CHVs and nurses will help achieve SDG 3 in terms of reducing maternal and under-five mortality. Evaluation of programs, confirming that home visits by CHVs and nurses reduce infant deaths and increase immunization take up across a range of situations continues to be important for low and middle income countries.

## Data statement

Data sets are available on request. The raw data that support the results and conclusions of the study can be made available, without undue reservation, to any qualified researcher.

## Author contributions

SK, AD, and EW conceived the study. SK, AD, EW, AS, and BM designed the study protocol and all authors read and approved the study protocol. AD and NH collected the data. SK, AD, AS, NH, and EW contributed to analyzing and interpreting the data. SK and AD drafted the manuscript and all authors critically revised the manuscript for intellectual content. All authors read and approved the final manuscript. SK and AD are guarantors of the paper.

### Conflict of interest statement

The authors declare that the research was conducted in the absence of any commercial or financial relationships that could be construed as a potential conflict of interest.
